# Risk factors for perioperative nerve injury associated with total knee arthroplasty: Analysis of a national administrative database

**DOI:** 10.1371/journal.pone.0324527

**Published:** 2025-06-02

**Authors:** Rahul H. Jayaram, Lucas Kim, Wesley Day, Rushabh H. Doshi, Lee E. Rubin, Jonathan N. Grauer

**Affiliations:** Department of Orthopaedics and Rehabilitation, Yale School of Medicine, New Haven, Connecticut, United States of America; Assiut University Faculty of Medicine, EGYPT

## Abstract

**Introduction:**

Nerve injury related to total knee arthroplasty (TKA) is a rare but serious complication. Previous studies identifying risk factors for nerve injury related to TKA have been constrained by institutional data or small cohorts. The current study utilized a comprehensive, national, administrative database to investigate independent risk factors for nerve injury associated with TKA.

**Materials and Methods:**

The PearlDiver M161 database was queried for adult TKA procedures performed between 2010 and 2022. Cases with postoperative nerve injury within 90 days of surgery were identified. Factors such as patient age, sex, body mass index (BMI), Elixhauser Comorbidity Index (ECI), fracture indication, and type of surgery (primary vs. revision) were evaluated for their correlation with nerve injury using multivariate analyses.

**Results:**

Out of 1,517,637 TKA procedures, nerve injury was identified for 4,480 (0.3%). Multivariate analysis identified the following independent risk factors for nerve injury, listed in decreasing order of odds ratio (OR): revision surgery (OR: 1.68), female sex (OR: 1.31), ECI ≥ 5 (OR: 1.27), and younger age (OR: 1.02 per decreasing decade) (*P* < 0.05 for each). Factors not significantly associated with nerve injury included underweight BMI (<20 kg/m^2^) and fracture indication. A decreased risk of nerve injury was observed in individuals with a BMI ≥ 35 kg/m^2^ (OR: 0.80, *P* = 0.002).

**Discussion:**

As expected, the incidence of nerve injury following TKA was low at 0.3%. Independent risk factors were identified for this adverse outcome, with the highest risk associated with revision surgeries. These findings, drawn from the largest cohort studied to date, offer valuable insights for risk stratification, and should inform patient discussions.

## Introduction

Total knee arthroplasty (TKA) is a prevalent orthopaedic procedure that is projected to grow in utilization over the years [1–3]. While TKA is generally regarded as a successful procedure with high satisfaction rates [4–7], significant adverse events, including nerve injuries, have been reported [8–13]. These nerve injuries can be debilitating and have a severe impact on patient outcomes, possibly resulting in lawsuit [8,14]. Recovery from these injuries can differ [9–11], as motor nerve injuries tend have a greater functional impact than sensory deficits [9]. Consequentially, it is essential to identify factors of TKA patients that increase susceptibility to nerve damage.

Prior analysis has found a low incidence of TKA-related nerve injury. One systematic review by Carender et al. found that in eleven studies including 47,585 TKAs, the incidence of postoperative peroneal nerve palsy was 0.4% [10]. A retrospective analysis of 383,000 TKAs from a New York State database found the incidence of nerve injury to be 0.12% [12], and another study from this database found the incidence in revision TKA to be higher at 0.56% [13]. Injuries to the peroneal nerve are most described in the literature [8,10,15]. However, injury has also been identified to other nerves of the lower extremity, including the sciatic and femoral nerves [12,16].

There have been numerous previous investigations to ascertain risk factors for nerve injury following TKA, with inconsistent results. In a retrospective review of 65 nerve injuries in 39,990 TKAs, Shetty et al identified female sex and a history of lumbar pathology as risk factors for nerve injury [16]. A different single-institution retrospective study found that patients with peroneal nerve palsy following TKA were younger and had higher BMI [17]. Moreover, female sex, younger age, in-hospital postoperative complications, valgus disorder, and previous spinal disorder were identified as risk factors in a statewide database study [12]. Nevertheless, these prior investigations were limited by either sample size or geographic distribution.

The purpose of the current study was to leverage a national, administrative claims database to produce a large sample to better determine the frequency and risk factors for nerve injury related to TKA.

## Materials and methods

### Study population

A retrospective analysis was performed using the M161 PearlDiver Ortho Mariner Patient Claims Database (PearlDiver Technologies, Colorado Springs, CO, USA) from 2010 to Q1 2022. This commercially available database contains an extensive collection of over 161 million patient records in the United States. With its vast repository of Health Insurance Portability and Accountability Act-compliant patient data, the PearlDiver database has gained increasing prominence in orthopaedic outcomes-based research [18–27]. As data from this database is in a de-identified and aggregated format, our Institutional Review Board (IRB) determined that studies utilizing this database are exempt from review. The authors did not have access to information that could identify individual participants during or after data collection.

The data used in this study are owned by the third-party vendor PearlDiver (URL: https://pearldiverinc.com/). The data can be queried using the Bellwether software, which is part of the PearlDiver database. The authors did not receive any special permissions or privileges beyond those granted through payment to the vendor.

TKA cases were queried using Current Procedural Terminology (CPT) code 27447 for primary procedures and CPT codes 27486, 27487, and 27488 for revision surgeries. The following exclusion criteria were then applied: age < 18 years; cases of infection or neoplasm; and missing 90-day follow-up healthcare data. Patient age, sex, body mass index (BMI), Elixhauser comorbidity index (ECI, a commonly used comorbidity index) [28], and indication for fracture were extracted from the dataset.

International Classification of Diseases (ICD)-9 and ICD-10 codes for nerve injury (refer to Table in S1 Table) were employed to find patients with nerve injuries within 90 days after TKA. Initial analyses indicated that the most frequent type of nerve injury involved unspecified codes. Given the challenge in clearly categorizing different nerve injuries and to increase study power, all nerve injury codes were collectively analyzed.

### Statistical analysis

Variables such as patient demographics, comorbidities, surgical indications, and types of surgery were compared between patients in the nerve injury and no-nerve injury cohorts. Univariable analyses using Pearson’s *chi*-squared test were conducted. Subsequently, multivariable logistic regression was performed for each variable to obtain odds ratios (ORs) and 95% confidence intervals (95% CIs).

PearlDiver’s internal software was used for statistical tests, with an alpha value set at 0.05. Figures were made using Microsoft Excel (Microsoft Corporation, Redmond, WA) and Graph-Pad Prism 9 (GraphPad Software, San Diego, CA).

## Results

The base cohort comprised of 1,517,637 patients who underwent TKA. Of these, nerve injury within 90 days of surgery was found for 4,480 (0.3%). Age, female sex, BMI status, ECI, and revision surgery were associated with nerve injury upon univariable analysis (Table 1).

**Table 1:** Univariate and Multivariate analysis of risk factors for nerve injury 90-days after TKA.

Variable	No Nerve Injury	%	Nerve Injury	%	*P*-value	Multivariate Odds Ratio with 95% CI		*P*-value
**Total**	1,513,157	99.7	4,480	0.3				
**Age (Per Decade Decrease)**	65.9 (18–84)		64.5 (23–83)		**<0.001**	1.02	(1.02, 1.02)	**<0.001**
**Sex**					**<0.001**			
Male	562,838	37.2	1,374	30.7		REF		
Female	950,316	62.8	3,106	69.3		1.31	(1.23, 1.40)	**<0.001**
**BMI**					**<0.001**			
< 20	15,502	1.0	65	1.5		1.01	(0.71, 1.42)	0.968
20-34	65,916	4.4	244	5.5		REF		
≥ 35 (Morbid Obesity)	510,207	33.7	1,735	38.7		0.80	(0.70, 0.92)	**0.002**
**ECI**					0.077			
0	126,298	8.3	322	7.2		REF		
1–2	367,760	24.3	1,021	22.8		1.09	(0.96, 1.24)	0.177
3–4	402,816	26.6	1,144	25.5		1.10	(0.97, 1.24)	0.145
≥5	157,377	10.4	523	11.7		1.27	(1.10, 1.46)	**<0.001**
**Fracture Indication**					0.138			0.618
No Fracture	1,509,282	99.7	4,463	99.6		REF		
Fracture	3,875	0.3	17	0.4		1.13	(0.70, 1.83)	
**Revision Surgery**					**<0.001**			**<0.001**
Non-Revision Case	1,393,307	92.1	3,887	86.8		REF		
Revision Case	119,850	7.9	593	13.2		1.68	(1.54, 1.84)	
BMI, Body Mass Index (kg/m^2)^								
ECI, Elixhauser Comorbidity Index								
Bold *P-value* = statistical significance at *P* < 0.05								

**Fig 1:**
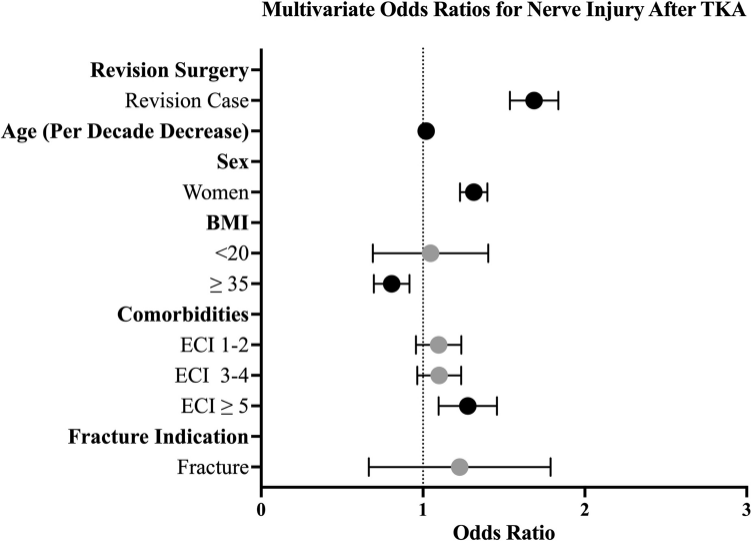
Forest Plot of Multivariate Odds Ratios for Risk Factors Associated with Nerve Injury 90 Days After Total Knee Arthroplasty.

Multivariable analyses found younger age (OR 1.02 for each decade decrease, P < 0.001), female sex (OR 1.31, P < 0.001), an ECI of 5 or higher (OR 1.27, P < 0.001), and revision procedure (OR 1.68, P < 0.001) to be significantly associated with nerve injury (Table 1, Fig 1).

Multivariable analysis also revealed significant non-associations with nerve injury, including underweight BMI (<20 kg/m2) and an indication of fracture. Conversely, a morbidly obese BMI (≥35 kg/m2) was associated with a reduced likelihood of nerve injury (OR 0.80, P = 0.002)

## Discussion

While TKA is regarded as a safe procedure, there is potential for nerve injury, which can have devastating consequences for patients, resulting in medical and legal ramifications [8,9]. The present study is the most extensive to date in evaluating the incidence and independent risk factors for nerve injury following TKA, drawing from 1,517,637 surgeries. From this population, nerve injuries were identified for 4,480 (0.3%), which is in the scope (0.12 to 1.3%) from previous literature [9,10,16,17,29,30]. While this is a low percentage of the overall cases, it represents a significant number of patients.

Numerous significant risk factors for nerve injury related to TKA were found. Of all variables examined, revision TKA (OR 1.68) carried the highest odds. This finding is expected, as revision surgeries typically present with a higher degree of scarring and anatomical variation. Comparative studies using prior statewide databases have highlighted this risk: Christ et al. reported a nerve injury incidence of 0.12% following primary arthroplasty [12], whereas Chen et al. found a higher incidence of 0.56% after revision TKA [13].

Age was another significant risk factor, with younger age carrying a higher odds (OR 1.02 per decade decrease) of nerve injury. While the specific reasons why younger patients are at higher risk for nerve injury after TKA cannot be defined here, this finding is consistent with prior literature [12,31]. Females were at higher odds of sustaining nerve injury after TKA than males (OR 1.31), which is also in line with prior literature [12,16]. Finally, an ECI score of greater than or equal to five indicated high patient comorbidity, was associated with greater odds of nerve injury (OR 1.27). This finding is supported by prior studies, which have found that patients with diabetes, tobacco use, and hypertension are at an increased risk of nerve injury following surgery [32].

Morbidly obese BMI status (≥35 kg/m^2^) was linked to decreased odds of post-operative nerve injury (OR 0.80), whereas underweight BMI status showed no relation. This finding conflicts with prior literature, which does not show an association between nerve injury and BMI range [11,16]. Given this, it is possible that the substantial patient population of this current study revealed an underlying association that would not be seen in smaller sample sizes. Obesity might be associated with greater soft tissue envelopes around structures that provides protection. Furthermore, the indication of fracture for TKA showed no association with nerve injury. To our knowledge, this relationship has not been previously evaluated, but it is encouraging to find that neither the fracture nor the surgical intervention appeared to increase the risk of nerve injury.

The current study is subject to limitations. Given its utilization of an administrative database, it depends on the accuracy of coding in administrative records. Additionally, specific details about patient anatomy and pathology are not accessible in this study, including the extent and volume of knee deformity being treated, the presence of soft tissue changes and contractures at the surgical site, and the type of neurologic deficit that occurs (partial vs. complete/motor vs. sensory). Furthermore, we were unable to assess the influence of surgical technique on post-operative neurological outcomes. Nevertheless, the large patient cohorts examined in this investigation have not been obtainable in previous research, significantly enhancing its statistical power.

Overall, the present study, involving over 1.5 million patients, is the largest investigation to date to analyze risk factors for nerve injury within 90 days of TKA. The incidence of nerve injury after TKA was low at 0.3%. Revision surgery, female sex, and ECI score of five or more were found to be associated with the greatest odds of nerve damage. Comprehending these risk factors can help future TKA patients by facilitating risk assessment, enhancing patient communication, and developing strategies to reduce risks during surgical planning.

## Supporting information

S1 Table International Classifications of Disease Codes and Descriptions of Nerve Injuries. (DOCX)
