# Supplementary material for: Risk factors for perioperative nerve injury associated with total knee arthroplasty: Analysis of a national administrative database
Source: PLoS One. 2025 Jun 2;20(6):e0324527. doi: 10.1371/journal.pone.0324527 (PMC12129142; doi:10.1371/journal.pone.0324527)
Supplement: S1 Table — (DOCX) [file pone.0324527.s001.docx]

| **Suppporting Table 1**: International Classifications of Disease Codes and Descriptions of Nerve Injuries | | |
| --- | --- | --- |
| **Affected Nerve** | **ICD Diagnosis Code** | **Description** |
| Sciatic Nerve | ICD-9-D-3550 | Lesion of sciatic nerve |
|  | ICD-9-D-9560 | Injury to sciatic nerve |
|  | ICD-10-D-G5700 | Lesion of sciatic nerve, unspecified lower limb |
|  | ICD-10-D-G5701 | Lesion of sciatic nerve, right lower limb |
|  | ICD-10-D-G5702 | Lesion of sciatic nerve, left lower limb |
|  | ICD-10-D-G5703 | Lesion of sciatic nerve, bilateral lower limbs |
|  | ICD-10-D-S7400XA | Injury of sciatic nerve at hip and thigh level, unspecified leg, initial encounter |
|  | ICD-10-D-S7400XD | Injury of sciatic nerve at hip and thigh level, unspecified leg, subsequent encounter |
|  | ICD-10-D-S7400XS | Injury of sciatic nerve at hip and thigh level, unspecified leg, sequela |
|  | ICD-10-D-S7401XA | Injury of sciatic nerve at hip and thigh level, right leg, initial encounter |
|  | ICD-10-D-S7401XD | Injury of sciatic nerve at hip and thigh level, right leg, subsequent encounter |
|  | ICD-10-D-S7401XS | Injury of sciatic nerve at hip and thigh level, right leg, sequela |
|  | ICD-10-D-S7402XA | Injury of sciatic nerve at hip and thigh level, left leg, initial encounter |
|  | ICD-10-D-S7402XD | Injury of sciatic nerve at hip and thigh level, left leg, subsequent encounter |
|  | ICD-10-D-S7402XS | Injury of sciatic nerve at hip and thigh level, left leg, sequela |
| Femoral Nerve | ICD-9-D-3552 | Other lesion of femoral nerve |
|  | ICD-9-D-9561 | Injury to femoral nerve |
|  | ICD-10-D-G5720 | Lesion of femoral nerve, unspecified lower limb |
|  | ICD-10-D-G5721 | Lesion of femoral nerve, right lower limb |
|  | ICD-10-D-G5722 | Lesion of femoral nerve, left lower limb |
|  | ICD-10-D-G5723 | Lesion of femoral nerve, bilateral lower limbs |
|  | ICD-10-D-S7410XA | Injury of femoral nerve at hip and thigh level, unspecified leg, initial encounter |
|  | ICD-10-D-S7410XD | Injury of femoral nerve at hip and thigh level, unspecified leg, subsequent encounter |
|  | ICD-10-D-S7410XS | Injury of femoral nerve at hip and thigh level, unspecified leg, sequela |
|  | ICD-10-D-S7411XA | Injury of femoral nerve at hip and thigh level, right leg, initial encounter |
|  | ICD-10-D-S7411XD | Injury of femoral nerve at hip and thigh level, right leg, subsequent encounter |
|  | ICD-10-D-S7411XS | Injury of femoral nerve at hip and thigh level, right leg, sequela |
|  | ICD-10-D-S7412XA | Injury of femoral nerve at hip and thigh level, left leg, initial encounter |
|  | ICD-10-D-S7412XD | Injury of femoral nerve at hip and thigh level, left leg, subsequent encounter |
|  | ICD-10-D-S7412XS | Injury of femoral nerve at hip and thigh level, left leg, sequela |
| Cutaneous Sensory Nerve | ICD-9-D-9564 | Injury to cutaneous sensory nerve, lower limb |
|  | ICD-10-D-S8420XA | Injury of cutaneous sensory nerve at lower leg level, unspecified leg, initial encounter |
|  | ICD-10-D-S8420XD | Injury of cutaneous sensory nerve at lower leg level, unspecified leg, subsequent encounter |
|  | ICD-10-D-S8420XS | Injury of cutaneous sensory nerve at lower leg level, unspecified leg, sequela |
|  | ICD-10-D-S8421XA | Injury of cutaneous sensory nerve at lower leg level, right leg, initial encounter |
|  | ICD-10-D-S8421XD | Injury of cutaneous sensory nerve at lower leg level, right leg, subsequent encounter |
|  | ICD-10-D-S8421XS | Injury of cutaneous sensory nerve at lower leg level, right leg, sequela |
|  | ICD-10-D-S8422XA | Injury of cutaneous sensory nerve at lower leg level, left leg, initial encounter |
|  | ICD-10-D-S8422XD | Injury of cutaneous sensory nerve at lower leg level, left leg, subsequent encounter |
|  | ICD-10-D-S8422XS | Injury of cutaneous sensory nerve at lower leg level, left leg, sequela |
| Peroneal Nerve | ICD-9-D-9563 | Injury to peroneal nerve |
|  | ICD-10-D-S8410XA | Injury of peroneal nerve at lower leg level, unspecified leg, initial encounter |
|  | ICD-10-D-S8410XD | Injury of peroneal nerve at lower leg level, unspecified leg, subsequent encounter |
|  | ICD-10-D-S8410XS | Injury of peroneal nerve at lower leg level, unspecified leg, sequela |
|  | ICD-10-D-S8411XA | Injury of peroneal nerve at lower leg level, right leg, initial encounter |
|  | ICD-10-D-S8411XD | Injury of peroneal nerve at lower leg level, right leg, subsequent encounter |
|  | ICD-10-D-S8411XS | Injury of peroneal nerve at lower leg level, right leg, sequela |
|  | ICD-10-D-S8412XA | Injury of peroneal nerve at lower leg level, left leg, initial encounter |
|  | ICD-10-D-S8412XD | Injury of peroneal nerve at lower leg level, left leg, subsequent encounter |
|  | ICD-10-D-S8412XS | Injury of peroneal nerve at lower leg level, left leg, sequela |
| Lateral Popliteal Nerve | ICD-9-D-3553 | Lesion of lateral popliteal nerve |
|  | ICD-10-D-G5730 | Lesion of lateral popliteal nerve, unspecified lower limb |
|  | ICD-10-D-G5731 | Lesion of lateral popliteal nerve, right lower limb |
|  | ICD-10-D-G5732 | Lesion of lateral popliteal nerve, left lower limb |
| Other/Unspecified | ICD-9-D-7289 | Unspecified disorder of muscle, ligament, and fascia |
|  | ICD-9-D-9565 | Injury to other specified nerve(s) of pelvic girdle and lower limb |
|  | ICD-9-D-9568 | Injury to multiple nerves of pelvic girdle and lower limb |
|  | ICD-9-D-9569 | Injury to unspecified nerve of pelvic girdle and lower limb |
|  | ICD-9-D-99709 | Other nervous system complications |
|  | ICD-10-D-S84801A | Injury of other nerves at lower leg level, right leg, initial encounter |
|  | ICD-10-D-S84801D | Injury of other nerves at lower leg level, right leg, subsequent encounter |
|  | ICD-10-D-S84801S | Injury of other nerves at lower leg level, right leg, sequela |
|  | ICD-10-D-S84802A | Injury of other nerves at lower leg level, left leg, initial encounter |
|  | ICD-10-D-S84802D | Injury of other nerves at lower leg level, left leg, subsequent encounter |
|  | ICD-10-D-S84802S | Injury of other nerves at lower leg level, left leg, sequela |
|  | ICD-10-D-S84809A | Injury of other nerves at lower leg level, unspecified leg, initial encounter |
|  | ICD-10-D-S84809D | Injury of other nerves at lower leg level, unspecified leg, subsequent encounter |
|  | ICD-10-D-S84809S | Injury of other nerves at lower leg level, unspecified leg, sequela |
|  | ICD-10-D-S8490XA | Injury of unspecified nerve at lower leg level, unspecified leg, initial encounter |
|  | ICD-10-D-S8490XD | Injury of unspecified nerve at lower leg level, unspecified leg, subsequent encounter |
|  | ICD-10-D-S8490XS | Injury of unspecified nerve at lower leg level, unspecified leg, sequela |
|  | ICD-10-D-S8491XA | Injury of unspecified nerve at lower leg level, right leg, initial encounter |
|  | ICD-10-D-S8491XD | Injury of unspecified nerve at lower leg level, right leg, subsequent encounter |
|  | ICD-10-D-S8491XS | Injury of unspecified nerve at lower leg level, right leg, sequela |
|  | ICD-10-D-S8492XA | Injury of unspecified nerve at lower leg level, left leg, initial encounter |
|  | ICD-10-D-S8492XD | Injury of unspecified nerve at lower leg level, left leg, subsequent encounter |
|  | ICD-10-D-S8492XS | Injury of unspecified nerve at lower leg level, left leg, sequela |
